# Characterization of Drug Resistance Mutations in *Mycobacterium tuberculosis* Isolates from Moroccan Patients Using Deeplex Targeted Next-Generation Sequencing

**DOI:** 10.3390/microorganisms13092163

**Published:** 2025-09-17

**Authors:** Said Laatri, Safaa El Kassimi, El Mehdi Bentaleb, My Driss El Messaoudi, Joy Irobi, Bouchra Belkadi, Abdelkarim Filali-Maltouf, Hassan Ait Benhassou

**Affiliations:** 1 Laboratory of Microbiology and Molecular Biology, Faculty of Sciences of Rabat, Mohammed V University, Rabat 10100, Morocco; 2 Prevention & Therapeutics Center, Moroccan Foundation for Advanced Science, Innovation and Research (MAScIR), Rabat 10100, Morocco; 3 Laboratory of Tuberculosis, Pasteur Institute of Morocco, Casablanca 20360, Morocco; 4 Biomedical Research Institute, Hasselt University, 3500 Hasselt, Belgium; 5 Prevention & Therapeutics Center, Moroccan Foundation for Advanced Science, Innovation and Research (MAScIR), Mohammed VI Polytechnic University (UM6P), Ben Guerir 43150, Morocco

**Keywords:** tuberculosis, *mycobacterium tuberculosis*, multidrug-resistant (MDR), extensively drug-resistant (XDR), targeted sequencing

## Abstract

Tuberculosis (TB) is a major public health concern worldwide and in Morocco, particularly considering the increasing burden of drug-resistant *Mycobacterium tuberculosis* (MTB) strains. In this study, we report the first nationwide molecular characterization of MTB clinical isolates using the Deeplex-MycTB targeted next-generation sequencing (tNGS) assay. A total of 71 culture-derived DNA samples from Moroccan TB patients were analyzed to detect resistance-associated mutations across 18 genes and to determine phylogenetic lineages. Of the 68 interpretable samples, 75% harbored either confirmed or uncharacterized mutations linked to drug resistance. Among these, 78% were classified as multidrug-resistant TB (MDR-TB) including 25.5% that met the criteria for pre-extensively drug-resistant TB (pre-XDR-TB). Mutations were most frequently identified in *rpoB*, *katG*, *inhA*, and *pncA*, consistent with resistance to rifampicin, isoniazid, and pyrazinamide. Phylogenetic analysis revealed a predominance of Lineage 4.3 (Euro-American) with a high representation of the LAM9 and T clades, some of which showed associations with specific resistance profiles. These findings highlight the utility of tNGS as a powerful tool for rapid resistance detection and molecular surveillance, with potential implications for guiding individualized treatment and informing national TB control strategies in Morocco.

## 1. Introduction

Tuberculosis (TB) is a global health threat with an estimated 10.8 million new cases and 1.25 million deaths in 2023 [1]. Although TB-related mortality has slightly declined since 2022, the global incidence continues to rise, driven in part by increasing drug resistance and post-pandemic diagnostic delays [1]. In Morocco, TB is endemic, with an incidence rate of 92 cases per 100,000 people in 2023, despite the implementation of a national control program based on DOTS and molecular diagnostics [2].

The emergence of drug-resistant TB strains presents an escalating challenge [1]. Approximately 400,000 people developed multidrug-resistant or rifampicin-resistant TB (MDR/RR-TB) in 2023, yet only 44% received appropriate treatment, leaving a substantial treatment gap [1]. Globally, MDR/RR-TB comprised 3.2% of new TB cases and 16% of previously treated cases in 2023, showing slight reductions from 2015 [1]. In Morocco, the situation is particularly concerning among retreatment cases, with 20% classified as pre-XDR and almost 7% as XDR [3]. Extensively, drug-resistant tuberculosis (XDR-TB) is defined as resistance to isoniazid and rifampicin (MDR-TB), as well as resistance to any fluoroquinolone and at least to bedaquiline or linezolid. Alarmingly, 19% of global MDR/RR-TB cases in 2023 exhibited fluoroquinolone resistance, reflecting substantial progress toward pre-XDR status [1,3,4]. Together, these data emphasize the urgent need to enhance TB diagnostic, treatment, and surveillance systems to mitigate the spread of drug-resistant *Mycobacterium tuberculosis* strains.

Traditional phenotypic drug susceptibility tests pose delays in the detection of resistance due to the slow growth rate (from days to weeks) when culturing *M. tuberculosis* specimens. Thus, rapid diagnosis of drug resistance to TB is crucial for the timely initiation of effective antibiotic treatment to prevent the spread of drug-resistant strains. Therefore, molecular-based techniques such as DNA sequencing, real-time PCR, DNA microarrays, and line probe assays, have been applied to detect mutations related to TB drug resistance using clinical samples or culture isolates [5,6,7,8].

The World Health Organization (WHO) has endorsed molecular line probe assays (LPAs), such as INNO-LiPA^®^ RIF TB, GenoType^®^ MTBDR/MTBDRplus, and GenoType^®^ MTBDRsl, the fully automated Xpert^®^ MTB/RIF assay, and recently, the Xpert^®^ MTB/RIF Ultra for the rapid determination of genetic mutations associated with resistance to rifampicin, isoniazid, and second-line anti-tuberculosis drugs [9,10,11]. Although these assays offer rapid analysis as well as high sensitivity and specificity, they have the disadvantage of being unable to cover a large panel of mutations into gene-conferring resistance and are expensive to implement in resource-limited countries.

More recently, next-generation sequencing (NGS) has emerged as a powerful tool in TB diagnostics, offering comprehensive insights into the genetic basis of drug resistance. By enabling the simultaneous detection of multiple resistance-associated mutations across the genome or within targeted gene panels, NGS provides a high-resolution approach to characterize DR-TB strains. Compared to conventional molecular tests, NGS delivers superior sensitivity and specificity and supports genomic surveillance efforts to track transmission dynamics and resistance trends [12,13,14]. More specifically, targeted NGS (tNGS) has proven effective for direct application on clinical specimens and isolates culture by targeting host spot regions. Recent studies have validated the utility of tNGS for comprehensive Drug Susceptibility Testing (DST), supporting its adoption as a complementary method in settings aiming to enhance diagnostic accuracy and precision [15,16,17].

In this study, we present the first nationwide genetic profiling of MTB strains collected from Moroccan patients. Using tNGS and especially Deeplex solution, the study objective is to characterize the spectrum of drug resistance mutations and provide insights into the circulating genetic lineages, thereby contributing relatively to the national TB control strategy through enhanced molecular surveillance and diagnosis of DR-TB.

## 2. Materials and Methods

### 2.1. Institutional Review Board Statement

The study was conducted in accordance with the Declaration of Helsinki, and the protocol was approved by the Ethics Committee of Biomedical Research, Rabat, Morocco (IRB00007914).

### 2.2. Informed Consent Statement

Informed consent was waived by the Ethics Committee of Biomedical Research, Rabat, Morocco, as the study involved the retrospective analysis of anonymized clinical samples collected during routine diagnostic procedures.

### 2.3. Samples Processing and LJ Culture

A total of 71 clinical MTB isolates were obtained from confirmed tuberculosis (TB) cases through the Tuberculosis Reference Laboratory at the Pasteur Institute of Morocco (Casablanca) between 2013 and 2016. Casablanca, as Morocco’s largest metropolitan city, reports the highest TB incidence in the country and serves as a major referral center.

The modified Petroff method was used for the decontamination process. Briefly, the sample was homogenized for 15 min in a shaker using an equal volume of NaOH (4%). The mixture was then centrifuged at 3000× *g* for 20 min. The resulting pellet was neutralized with 20 mL of sterile distilled water, followed by a second centrifugation step. Aliquots of decontaminated specimens were inoculated in duplicate into LJ medium. Media were then incubated at 37 °C for up to 4 weeks. All isolates were identified as MTB by their slow growth rate, colony morphology, and colony pigmentation.

### 2.4. Phenotypic Drug Susceptibility Testing (pDST):

pDST was performed retrospectively at the Moroccan National Reference Laboratory on Löwenstein–Jensen medium using the proportion method but was routinely available only for rifampicin (RIF) and isoniazid (INH), the two key first-line anti-tuberculosis drugs. Critical concentrations applied were 40 μg/mL for RIF and 0.2 μg/mL for INH, following WHO recommendations. For other anti-tuberculosis drugs, resistance prediction relied exclusively on genotypic data obtained using the Deeplex-MycTB assay (Genoscreen, Lille, France), complemented when available by whole-genome sequencing (WGS). Resistance-associated variants were interpreted based on the WHO Catalog of mutations in MTB [1].

### 2.5. Genomic DNA Extraction

Genomic DNA was extracted from cultured MTB isolates using the QIAamp DNA Mini Kit (Qiagen, Germantown, MD, USA) following the manufacturer’s protocol. DNA concentration and purity (A260/A280) were assessed with NanoDrop 2000 (Thermo Fisher Scientific, Waltham, MA, USA), and DNA quantification was performed with the Qubit dsDNA BR Assay Kit (Life Technologies, Paisley, UK). DNA integrity was confirmed using the Agilent Bioanalyzer (Agilent Technologies, Santa Clara, CA, USA). Only DNA samples presenting optimal purity (A260/A280 between 1.8 and 2.0) and intact profiles were retained for downstream applications. DNA was stored at −20 °C until use.

### 2.6. Targeted Sequencing and Library Preparation

Targeted sequencing was carried out using the Deeplex^®^-MycTB assay (GenoScreen), a multiplexed PCR-based method that amplifies 24 genomic regions in a single reaction. The assay targets 18 genes associated with drug resistance in the *M. tuberculosis* complex (MTBC) along with the *hsp65* gene for Mycobacterial speciation and the DR/CRISPR locus for spoligotyping. Notably, two separate amplicons cover *rpoB* codons 384–516 and 158–335, encompassing the rifampicin resistance-determining region (RRDR) and other loci known to harbor resistance-conferring mutations. For *ethA*, two overlapping amplicons span the coding sequence and a portion of the upstream promoter. For genes like *pncA* and *Rv0678*, which are, respectively, linked to pyrazinamide and bedaquiline/clofazimine resistance, a single amplicon covers the entire coding region and part of the promoter.

Amplicons were purified using AMPure XP magnetic beads (Beckman Coulter, Brea, CA, USA) and quantified with the Qubit dsDNA High-Sensitivity Assay Kit on a Qubit 3.0 Fluorometer (Thermo Fisher Scientific). Sequencing libraries were prepared using the Nextera XT DNA Library Preparation Kit (Illumina Inc., San Diego, CA, USA), generating paired-end libraries with a read length of 150 bp. Library quality was verified using the High-Sensitivity DNA Kit on an Agilent Bioanalyzer (Agilent Technologies). Equimolar pooling of libraries was followed by sequencing on the Illumina MiSeq platform using standard procedures.

### 2.7. Bioinformatic Analysis and Interpretation of Drug Resistance

Prior to analysis, the quality of raw sequencing reads was assessed using FastQC (Babraham Institute, UK) to evaluate key quality metrics, including per-base Phred scores, GC content, and read length distribution. Only high-quality datasets that passed these checks were retained for downstream analysis.

Raw reads were analyzed using the Deeplex^®^-MycTB cloud-based analytical platform (GenoScreen; version 2), a dedicated tool for the standardized analysis and interpretation of tNGS data for MTB. The platform predicts drug resistance profiles based on curated reference datasets, including ReSeqTB and the WHO Catalog of Mutations in *MTB* Complex (1). Variant calling was performed using a validated internal pipeline with predefined quality control thresholds. Only bases with a minimum Phred quality score of 30, corresponding to a base call accuracy of ≥99.9%, were considered for variant detection.

Mutations were classified as fixed when present in ≥97% of reads at a given locus, and as unfixed when present in ≥3% but <97% of reads, reflecting potential heteroresistance. A minimum read depth of 100× per targeted locus was required for confident variant calling. Variants not listed in established resistance mutation catalogs were categorized as “uncharacterized variants” and systematically reported for transparency.

## 3. Results

### 3.1. Sequencing Quality and Sample Selection

Two successive sequencing runs were performed on 48 samples including 3 controls, using the Illumina MiSeq platform with V2 flow cells (Illumina Inc.,). Due to suboptimal quality scores in the first run, 19 samples were re-sequenced in the second run. For each of these, the dataset with the higher sequencing quality was retained for downstream analysis.

**Figure 1:**
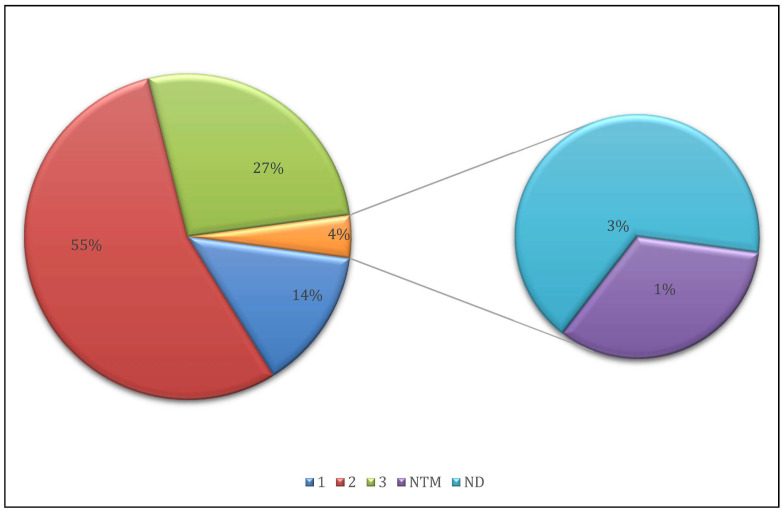
The proportion of sequencing quality scores on 71 sequenced clinical samples. 1: All resistance-associated positions in the database with enough data to identify mutations from 80 to 100%; 2: All resistance-associated positions in the database with enough data to identify mutations from 10 to 100%; 3: All resistance-associated positions in the database with enough data to identify mutations from 3 to 100%; ND: Mycobacteria not detected; NTM: Non-tuberculous mycobacteria detected.

Run performance metrics showed that the percentage of clusters passing filter (PF) was 96.68% and 93.06% for runs 1 and 2, respectively, while the proportion of reads with Phred quality scores above Q30 reached 95.46% and 92.92%, respectively. FastQ files were generated for all samples and uploaded to the GenoScreen web application for automated processing. The platform assigned a sequencing quality score ranging from 1 to 3 for each sample based on data completeness and depth. Out of the 71 total samples, 39 (54.9%) were rated as quality score 2, 19 (26.8%) as score 3, and 10 (14.1%) as score 1. Three samples, 44, 49, and 51, were excluded from the final analysis due to failed MTB detection (samples 49 and 51) or the presence of non-tuberculous Mycobacteria (sample 44). Consequently, the final study cohort comprised 68 clinical samples (Figure 1).

### 3.2. Mapping and Coverage Statistics

**Figure 2:**
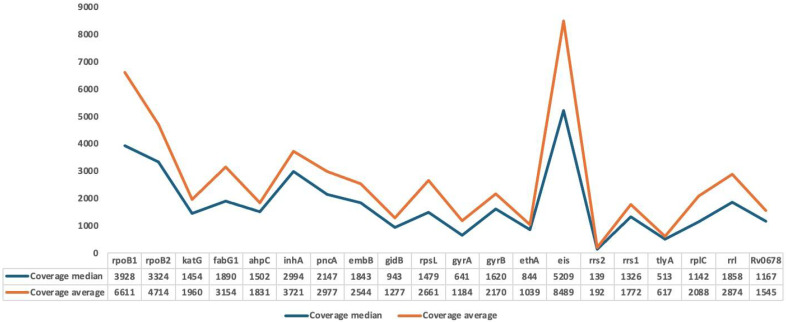
Coverage depth per targeted gene in 71 sequenced clinical *Mycobacterium tuberculosis* isolates.

Sequence reads were aligned to the *M. tuberculosis H37Rv* reference genome using the GenoScreen pipeline. The mean genome coverage breadth across the 68 samples was 99.8%, indicating near-complete representation of the targeted genomic regions (Supplementary Table S1). Depth of coverage ranged from 267× to 32,724×, with a mean depth of 4123× and a median of 2263×. The three excluded samples exhibited insufficient median coverage (<50×), supporting their exclusion. Gene-specific coverage analysis showed the *rrs* gene had the lowest median coverage (139×) while the *eis* gene had the highest (5209×), with an overall median coverage of 1798× across all targeted genes (Figure 2, Supplementary Table S2).

### 3.3. Drug Resistance Profiles

Among the 68 sequenced MTB clinical isolates, 17 (25%) were fully susceptible, with no resistance-associated or uncharacterized variants detected (Supplementary Table S3). The remaining 51 isolates (75%) harbored resistance-associated variants (RVs), with or without additional uncharacterized variants (UVs). Among these, 40 isolates (78.4%) were classified as multidrug-resistant tuberculosis (MDR-TB), including 13 isolates (32.5%) meeting the definition of pre-extensively drug-resistant TB (pre-XDR-TB). An additional 7 isolates (13.7%) carried RVs conferring resistance to one or more drugs without fulfilling the criteria for MDR or pre-XDR. In total, 47 isolates were considered drug-resistant based on the presence of RVs, affecting 11 out of the 18 resistance-associated genetic targets covered by the Deeplex-MycTB assay.

### 3.4. Resistance by Drug Class

Resistance to first-line drugs, as defined by the WHO (rifampicin, isoniazid, pyrazinamide, and ethambutol), was commonly detected. Five distinct mutations were identified in the *rpoB* gene among 40 rifampicin-resistant isolates, with S450L being the most prevalent (31/40; 77.5%). For isoniazid, the S315T mutation in *katG* was detected in all 42 resistant isolates, while the C-15T mutation in *fabG1* (genomic position 1,673,425) was present in 16 isolates. No RVs were observed in *ahpC* or *inhA*. Pyrazinamide resistance was linked to nine mutations in *pncA* and detected in 18 isolates, with D8E (5 cases) and a deletion at position 2,289,010 (6 cases) being the most frequent. Ethambutol resistance was associated with six *embB* mutations found in 28 isolates, including M306V (10 isolates) and Q497K (9 isolates).

For WHO Group A drugs (fluoroquinolones, bedaquiline, and linezolid), resistance was observed in 13 isolates carrying mutations in *gyrA*. Seven distinct mutations were identified in this gene, with D49G being the most common (4 cases). These 13 isolates were classified as pre-XDR-TB, based on resistance to both rifampicin and a Group A drug (fluoroquinolone). No resistance-associated mutations were identified in *rrl, rplC*, *Rv0678*, or *atpE*, and no confirmed mutations linked to bedaquiline, clofazimine, or linezolid resistance were detected.

**Figure 3:**
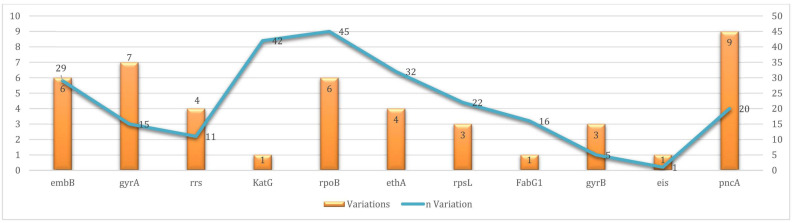
Prevalence of resistance variations per targeted gene in 68 sequenced clinical Mycobacterium tuberculosis isolates.

Among WHO Group C drugs, streptomycin resistance was conferred by three mutations in *rpsL*, with K88R and K43R detected in 13 and 8 isolates, respectively. Ethionamide resistance was associated with four mutations in *ethA*, found in 32 isolates, including a delCACGTCG event in 15 isolates and a deletion at genomic position 4,327,367 in 10 isolates. The C-14T mutation in *eis*, which is associated with low-level kanamycin resistance, was observed in one isolate. Seven UVs were identified in the *rrs* gene in a single isolate, but due to low sequencing depth, resistance to high-level injectable agents such as amikacin and capreomycin could not be confirmed in this case (Figure 3, Table 1 and Table 2).

### 3.5. Uncharacterized Variants (UVs)

**Figure 4:**
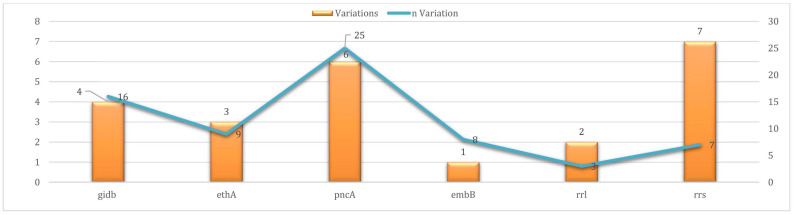
Prevalence of uncharacterized variations per targeted gene in 68 sequenced clinical Mycobacterium tuberculosis isolates.

A total of 23 uncharacterized variants were identified across six resistance-associated genes: *pncA*, *embB*, *gidB*, *ethA*, *rrs*, and *rrl.* In *pncA,* six distinct UVs were found in 25 isolates (36.8%), with T142R being the most frequently observed (14 isolates), followed by V163G and C72Y (3 isolates each), D49Y and H57Q (2 isolates each), and A171T in 1 isolate. The *embB* gene harbored a single UV, N296H, which was consistently found in eight isolates. In *gidB,* four UVs were detected in 16 isolates, with Y22Stop being identified in 7 cases and inserC4407856 in 6. The *ethA* gene contained three UVs found in nine isolates, including Q271Stop in five of them. Seven UVs were also observed in the *rrs* gene within a single isolate, although low sequencing depth limited interpretation. Finally, two UVs were identified in the *rrl* gene among three isolates, at genomic positions 1,476,007 and 1,476,251. These *rrl* mutations have not been definitively associated with alterations in ribosomal function or with linezolid resistance. Most of the UVs were associated with genes linked to resistance to Group C drugs, particularly ethionamide, streptomycin, and aminoglycosides (Figure 4, Table 3).

### 3.6. Concordance Between Phenotypic and Genotypic Testing

Phenotypic drug susceptibility testing (pDST) was performed in parallel with tNGS for all 68 isolates. A perfect concordance (100%) was observed for rifampicin and isoniazid resistance. All 40 isolates with *rpoB* mutations were phenotypically resistant to rifampicin, and all 42 isolates carrying mutations in *katG* and/or *fabG1* were phenotypically resistant to isoniazid. These results confirm the strong predictive value of genotypic testing for first-line drug resistance in this cohort (Table 4).

### 3.7. Statistical Analysis of Variant Distribution

Chi-square (Chi^2^) tests were conducted to assess the distribution patterns of RVs and UVs across genes and drug categories. A significant difference was found in the distribution of RVs and UVs at the gene level (Chi^2^ = 110.6; *p* < 0.0001). Genes such as *katG*, *pncA*, and *embB* showed the highest frequency of RVs, while *pncA*, *embB*, and *ethA* carried the highest burden of UVs. A statistically significant difference was also observed at the drug group level when comparing first-line, XDR-related, and Group C drugs (Chi^2^ = 7.53; *p* = 0.023). First-line drugs were predominantly associated with RVs, whereas Group C drugs showed a notable accumulation of UVs.

A highly significant association was observed between mutation type and gene (Chi^2^ = 958.8; *p* < 0.0001). Certain mutations were strongly gene-specific, including S315T in *katG* (42 isolates), S450L in *rpoB* (31 isolates), delCACTGGT in *ethA* (15 isolates), and K88R in *rpsL* (13 isolates). This gene-specific clustering of mutations supports the evolutionary and functional relevance of these canonical variants.

### 3.8. Phylogenetic Analysis and Lineage Distribution

**Figure 5:**
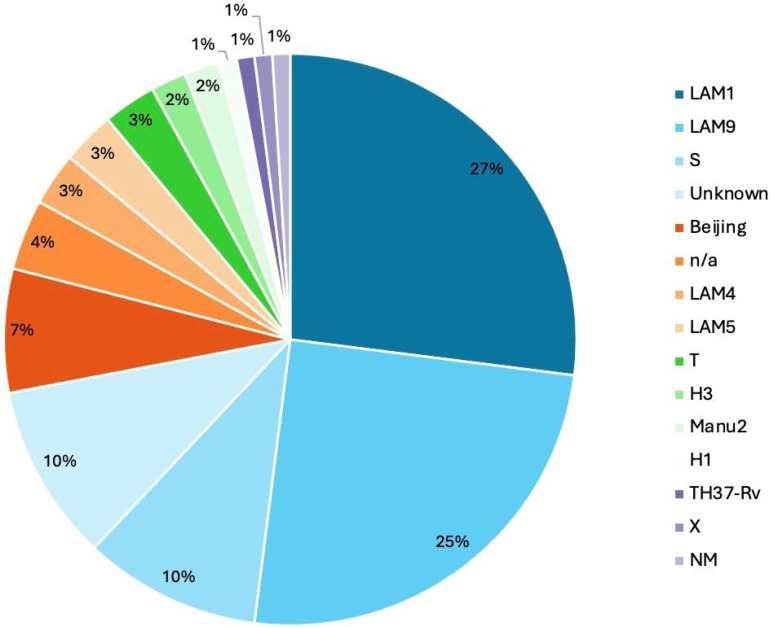
The proportion of classification by the lineage of *Mycobacterium tuberculosis* isolates from 68 sequenced clinical samples.

**Figure 6:**
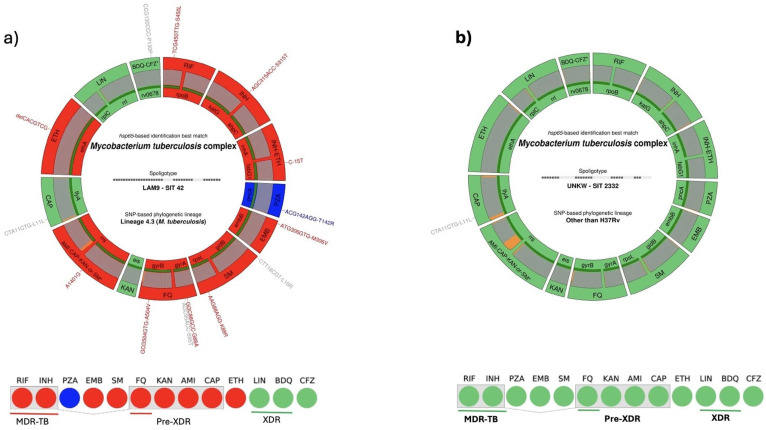
Drug resistance and lineage profiles of two Mycobacterium tuberculosis clinical isolates from Moroccan patients by the WHO-endorsed tNGS assay, Deeplex-MycTB (GenoScreen). Circular plots summarize for each isolate the species identity, spoligotype, phylogenetic lineage, and resistance-associated mutations across 13 anti-tuberculosis drugs. Each concentric ring represents one drug, with resistance-conferring mutations highlighted in red (high-confidence), blue (intermediate-confidence), and green (susceptible/wild-type). Bottom legends indicate MDR, Pre-XDR-TB, and XDR classification status. (**a**) Isolate belonging to the LAM9 sublineage (SIT 42, Lineage 4) shows mutations conferring resistance to RIF, INH, PZA, EMB, SM, FQ, KAN, and AMI, classifying it as Pre-XDR-TB. (**b**): Fully susceptible isolates of T1 (SIT 33) lineage, with no mutations detected in any drug-resistant genes.

SNP-based phylogenetic analysis of the 68 clinical MTB isolates revealed 15 distinct spoligotype-defined clades. The most prevalent were clades T (26.8%) and LAM9 (25.4%), followed by clade S (9.9%). The Beijing clade was observed in 5 isolates (7.4%), while clades H1, H3, and Manu2 were each found in 2 isolates (2.9%). Single isolates belonged to the X, LAM1, LAM4, LAM5, and H37Rv clades (1.5% each). Seven isolates could not be conclusively assigned to a spoligotype-defined clade due to incomplete lineage-defining mutations (Figure 5 and Figure 6 Among the same cohort, 11 potential mixed infections (16.2%) were identified based on the detection of lineage-defining mutations with variant allele frequencies below 95% (Table 5).

At the lineage level, 32 isolates (47.1%) were classified as L4.3 (Euro-American), 6 (8.8%) as L2 (Beijing), and 30 (44.1%) as belonging to other/unassigned lineages (Figure 5, Table 6).

### 3.9. Clade–Lineage Correlation in Drug-Resistant Samples

Among the 40 multidrug-resistant (MDR) isolates, clades T and LAM9 were most frequently observed, accounting for 12 (30.0%) and 11 (27.5%) samples, respectively. Other clades included S (12.5%), Beijing (10.0%), and Manu2 (5%). Two MDR samples had unknown clade classification, and one was unassigned. Within the MDR group, 13 isolates (32.5%) met the criteria for Pre-XDR. In this Pre-XDR subset, lineage–clade correlation showed that 5 (38.5%) belonged to the L4.3/LAM9 group and 4 (30.8%) to the S clade, with no single spoligotype or lineage predominating.

Chi^2^ tests revealed no statistically significant association between SNP-defined lineages and either MDR status (χ^2^ = 2.31, *p* = 0.315) or Pre-XDR status (χ^2^ = 1.95, *p* = 0.377). Observed lineage proportions deviated only slightly from expected values under the null hypothesis. Similarly, analysis of spoligotype-defined clades showed that MDR prevalence exceeded 84% across all major clades and reached 100% in Beijing, LAM1, H1, and Manu2. In contrast, the prevalence of Pre-XDR remained low overall, with the highest frequency observed in the LAM9 clade (~7.7%). Bar plots further confirmed that resistance to fluoroquinolones and Group A/B drugs (LIN/BDQ/CFZ) was sporadic across clades, supporting the absence of any dominant Pre-XDR-associated lineage or clade (Figure 6, Table 5 and Table 7, Supplementary Table S4).

## 4. Discussion

Our study provides a comprehensive molecular characterization of MTB using the Deeplex-MycTB targeted sequencing panel. The sequencing runs achieved high-quality metrics (clusters passing filter >93%, Q30 >92%), which translated into exceptional genome coverage breadth (99.8%) and depth (median XP ~2263×). This depth allowed confident detection of low-frequency alleles and mixed infections, aligning with previous targeted sequencing validation efforts. Three samples were excluded due to poor mapping metrics, reinforcing the need for strict QC in clinical sequencing workflows [18].

With 71 clinical isolates analyzed, including 68 passing rigorous quality control (QC), we observed a high prevalence of drug resistance associated with both canonical mutations and novel variants. For instance, in the *rpoB* gene, five distinct mutations were identified across 40 MTB isolates. The most prevalent was the S450L substitution, detected in 31 samples (77.5%) which is consistent with our earlier findings on the same cohort using High-Resolution Melting (HRM) analysis followed by Sanger sequencing confirmation [19]. Bentaleb et al. (2017) [18] study specifically targeted the most commonly mutated codons within the rifampicin resistance-determining region (RRDR), namely codons 516, 526, and 531. However, among the 45 phenotypically rifampicin-monoresistant isolates confirmed by Löwenstein–Jensen (LJ) culture, five were identified as genotypically wild-type by HRM, thus reducing the sensitivity of the HRM assay [19]. Indeed, the Deeplex-based tNGS employed in the present study successfully resolved these discrepancies, identifying 5 additional mutations: H445Y (*n* = 3) and V170F (*n* = 2). Notably, the V170F mutation, although located outside the RRDR, is a well-documented contributor to discordant rifampicin resistance cases, as previously reported by Solari et al., 2020 [19], and Merker et al., 2021 [20]. Globally, V170F was observed in approximately 1–2% of RIF-resistant strains, indicating a relatively low but regionally significant prevalence [21,22]. To our knowledge, this is the first report to describe the V170F mutation in clinical isolates from Morocco. Its detection highlights the need for expanded molecular surveillance to assess its distribution and evolutionary dynamics within the region. Large-scale, geographically diverse studies will be essential to elucidate the prevalence and potential public health impact of this SNP in North African TB settings.

Moreover, several resistance-associated mutations were identified in MTB clinical isolates. Most notably, the N296H substitution in the *embB* gene was frequently observed and is a well-documented mutation associated with resistance to ethambutol (EMB) [23]. This mutation has been classified as “Associated with Resistance” by the WHO mutation catalog and is frequently detected in clinical EMB-resistant strains [24]. Similarly, multiple nonsynonymous mutations were detected in the *pncA* gene, including T142R, V163G, A171T, D49Y, H57Q, and C72Y. These mutations are distributed throughout the gene, which is consistent with the known high diversity of *pncA* mutations conferring pyrazinamide (PZA) resistance, as outlined in several studies [25,26] and listed in the WHO catalog. PZA plays a pivotal role in standard TB regimens by targeting persistent bacilli; resistance undermines treatment efficacy and prognosis. Our data revealed a broad spectrum of *pncA* mutations, including several previously reported and others classified as uncharacterized (UVs).

The diversity and gene-specific distribution of uncharacterized variants were further analyzed statistically, revealing a significant difference across resistance-associated genes (Chi^2^ = 12.83, *p* = 0.025), with *pncA* and *rrs* harboring the highest number of UVs. Moreover, the overall gene–mutation specificity was confirmed by a highly significant chi-square test (Chi^2^ = 958.8; *p* < 0.0001), supporting a non-random and functionally structured evolution of resistance.

The recurrence of these UVs, particularly within key resistance determinants, raises critical questions about their biological significance. While their clinical impact remains uncertain, their consistent presence in resistance-associated loci suggests they may either reflect emerging resistance mechanisms, compensatory mutations, or lineage-specific polymorphisms. The WHO mutation catalog emphasizes that such variants should not be automatically linked to resistance without robust phenotypic or functional validation [24]. Nevertheless, their repeated detection across studies, including ours, highlights a potential gap in current reference databases and underscores the need for systematic reporting. Importantly, the high prevalence of UVs in *pncA* mirrors the gene’s intrinsic mutational diversity, known to complicate the molecular prediction of pyrazinamide resistance. This phenomenon may partially explain discordances observed between genotypic and phenotypic testing in various settings [27,28,29,30]. Similarly, UVs in genes like *gidB*, *rrs*, and *rrl*, although less frequent, warrant further investigation, especially considering their potential to contribute to low-level resistance or diagnostic ambiguity.

The L397P mutation in *ethA*, a gene involved in the activation of ethionamide (ETH), was also detected and is included in the WHO database as a resistance-associated variant. Mutations in *ethA* can lead to loss of prodrug activation, thereby conferring ETH resistance [31]. In addition, multiple mutations in *gidB* (e.g., L44P, Y22Stop, and insertC) were observed. These mutations are frequently associated with low-level resistance to streptomycin (SM) and have been previously reported in resistant strains, though often not sufficient alone to confer high-level resistance [24,32].

Furthermore, some mutations identified in *rrs* (e.g., A1278T and insertions) and *rrl* (T2350G, T2594C) were not listed in the WHO catalog. These positions lie outside the canonical hotspots known for aminoglycoside resistance (such as A1401G, C1402T, and G1484T in *rrs*) and thus require further investigation. Their clinical relevance remains uncertain and should be validated through functional assays or MIC correlation studies.

Several of these insertions and deletions, such as insertC, delC, and delCACTGGT, detected in *pncA*, *ethA*, and *rrs*, represent structural variations that may be overlooked in standard SNP-based diagnostics. Their presence highlights the need to expand predictive tools and leverage approaches such as CRISPR interference or deep mutational scanning to better understand their phenotypic impact.

Notably, all resistance-associated mutations identified by Deeplex-MycTB were also detected by a whole-genome sequencing (WGS) conducted independently on the same set of isolates, demonstrating complete concordance between the two methods. This strong agreement underscores the reliability of targeted sequencing not only for detecting resistance but also for confidently identifying uncharacterized or rare mutations. The raw WGS reads supporting this validation are publicly available in the NCBI Sequence Read Archive under BioProject accession number PRJNA1048908.

In our study, 47 out of 68 MTB isolates (69.1%) harbored resistance-associated variants (RVs) and were therefore classified as drug-resistant. This group included 40 multidrug-resistant (MDR) isolates (58.8%), of which 13 (32.5% of MDR cases) met the criteria for pre-extensively drug-resistant TB (pre-XDR-TB), and 7 isolates (10.3%) carried RVs conferring resistance to one or more drugs without meeting MDR or pre-XDR definitions. These drug-resistant variants affected 11 out of the 18 resistance-associated genetic targets covered by the Deeplex-MycTB assay. In several cases, RVs co-occurred with uncharacterized variants (UVs), whose clinical impact remains uncertain. While UVs were not used for resistance classification, their frequent co-detection with RVs in key resistance loci highlights the importance of ongoing catalog updates and functional validation efforts.

In our study, mixed MTB infections were detected in 16.2% of clinical samples, a prevalence consistent with reports from high-burden settings (Table 8) [33,34]. Mixed infections (defined as co-infection with genetically distinct MTB strains within the same host) pose important clinical and public health challenges. They may involve both drug-susceptible and drug-resistant strains, potentially undermining the efficacy of standard treatment regimens and increasing the risk of treatment failure, relapse, or acquired resistance due to selective pressure during therapy [34]. Additionally, the presence of minority resistant subpopulations can compromise both molecular and phenotypic drug susceptibility testing (DST), leading to false-negative results and inappropriate treatment decisions [34]. A recent study from Uganda demonstrated that mixed infections could reduce the sensitivity of widely used molecular assays such as GeneXpert, further complicating the clinical management of MDR-TB [35]. These infections may also modulate host immune responses, increase susceptibility to reinfection, and alter disease progression dynamics [36,37].

Given these complexities, the ability to detect mixed strain infections is essential for accurate diagnosis and tailored treatment. Traditional genotyping tools such as spoligotyping and MIRU-VNTR have historically provided insights into strain diversity and transmission patterns, with MIRU-VNTR being widely used for identifying mixed infections [38]. However, targeted deep sequencing technologies like the WHO-endorsed Deeplex-MycTB assay offer superior resolution, enabling detection of minority variants at frequencies as low as 1%. In our study, this approach proved instrumental in uncovering heterogeneous mutations in *pncA* and other genes, suggesting the coexistence of pyrazinamide-resistant and -susceptible bacilli within the same host. Such heterogeneity has significant clinical implications, as it may compromise treatment efficacy and facilitate the selection of resistant clones under drug pressure [24,39,40,41]. Although the retrospective design of our study precluded assessment of clinical outcomes, our findings underscore the relevance of high-resolution sequencing for both clinical and surveillance purposes. Future research should focus on systematically mapping mixed infection patterns across regions and timeframes in Morocco to better understand transmission dynamics and support targeted public health interventions.

The use of targeted deep sequencing in our analysis was instrumental in detecting minority variants with frequencies as low as 1%, revealing genetic heterogeneity that would likely have been missed by conventional DST or genotyping methods. In particular, we observed heterogeneous *pncA* mutations in several mixed infections, suggesting the simultaneous presence of both PZA-resistant and PZA-susceptible bacilli. This scenario complicates clinical decision-making, as standard regimens may not fully eradicate resistant subpopulations, thereby increasing the likelihood of selection under drug pressure [39,40].

The high concordance observed between phenotypic drug susceptibility testing (pDST) and targeted next-generation sequencing (tNGS) for rifampicin and isoniazid resistance in our study underscores the clinical relevance of mutations in *rpoB*, *katG*, and *fabG1* genes. All isolates harboring mutations in these targets exhibited phenotypic resistance, supporting their role as key molecular markers for first-line drug resistance detection. These findings align with earlier reports showing a strong agreement between molecular detection of *rpoB* mutations and phenotypic rifampicin resistance [3,4,5]. Likewise, the frequent occurrence of the *katG S315T* mutation among isoniazid-resistant strains confirms its predictive value, as emphasized in the WHO mutation catalog and studies on isoniazid and ethionamide resistance [23,30]. Additionally, mutations in the *fabG1-inhA* promoter region, associated with low-level isoniazid resistance, further support their inclusion in resistance prediction panels [30]. Our data strengthen the role of the Deeplex-MycTB assay as a reliable tool for rapid detection of first-line drug resistance in clinical practice.

Of the 68 MTB isolates investigated in this study, 47 (69.1%) harbored resistance-associated variants (RVs) and were thus classified as drug-resistant. This included 40 MDR-TB isolates and 13 that met the pre-XDR criteria. Lineage analysis revealed a predominance of Lineage 4.3, with the LAM and T subclades collectively representing 69% (*n* = 36/52) of all MDR/pre-XDR isolates. The LAM9 sublineage was particularly overrepresented, suggesting local clonal expansion of resistant strains in Morocco.

However, statistical analysis failed to identify any significant association between lineage (or clade) and MDR or pre-XDR classification, suggesting that resistance acquisition in this setting is not confined to specific phylogenetic backgrounds. These results are in line with recent WHO reports [1] and studies such as Merker et al. [20] which suggest that drug resistance can emerge independently across lineages, influenced more by treatment pressure and antibiotic availability than by lineage-specific mechanisms.

Notably, only a small proportion of the sequenced isolates (5 out of 68; 7.4%) belonged to the Beijing lineage, reflecting its limited circulation in Morocco. This low prevalence contrasts with global reports associating Beijing strains with increased transmissibility and drug resistance, particularly in Asia and Eastern Europe. Our findings are consistent with previous molecular epidemiological studies conducted in Morocco, which have repeatedly shown the predominance of Euro-American lineages, especially LAM and T families, and only sporadic detection of Beijing strains. For instance, Lahlou et al. [42] and Chaoui et al. [43] reported LAM as the most prevalent lineage in both drug-susceptible and resistant TB isolates, while Beijing strains were rarely observed [42,43]. More recently, Oudghiri et al. confirmed this trend among MDR and pre-XDR isolates from Casablanca, highlighting the genotypic diversity within LAM sublineages and the persistent underrepresentation of Beijing strains in the Moroccan context [44].

Although our data contribute to the understanding of drug resistance dynamics in Morocco, several limitations must be acknowledged. First, the isolates analyzed were collected between 2013 and 2016. While this period provided a valuable historical snapshot, it may not fully reflect recent trends in drug resistance evolution, especially with the emergence of bedaquiline and linezolid resistance. Continuous and updated surveillance efforts are needed to assess the current landscape and track the emergence of new resistance patterns.

Second, although sequencing coverage was generally excellent across most targets, certain loci such as *rrs* exhibited relatively lower coverage. This could have limited our ability to detect low-frequency variants or structural mutations within these genes, which are important for resistance to aminoglycosides and capreomycin. Ensuring uniform target enrichment across all loci remains a technical challenge in amplicon-based sequencing platforms.

Third, our study was conducted using isolates collected at a single reference center in Casablanca, Morocco’s largest urban area and a national TB hotspot. While Casablanca reflects a high-incidence context, it may not capture the full diversity of circulating strains and resistance profiles across other Moroccan regions, including rural and peripheral settings. Moreover, as a tertiary referral center, there is potential for selection bias toward more severe or drug-resistant cases. Therefore, multicenter and prospective sampling approaches are warranted to validate our findings at the national level.

Finally, although the Deeplex-MycTB platform is a powerful tool, its interpretive capacity remains partly limited by the scope and completeness of existing mutation catalogs. As shown in our study, several recurrent mutations, particularly uncharacterized variants (UVs) and structural alterations, remain unclassified in WHO and other databases, highlighting an urgent need for curated region-specific entries and high-throughput functional validation pipelines.

## 5. Conclusions

Our study highlights the utility of tNGS for comprehensive characterization of drug resistance and strain diversity among MTB isolates in Morocco. Beyond its diagnostic value, tNGS holds promise for refining treatment strategies and enhancing surveillance. However, the broader implementation of such approaches in resource-limited settings like Morocco remains challenged by cost, infrastructure, and workforce training requirements. Coordinated efforts are needed to reduce costs, develop accessible platforms, and strengthen molecular surveillance capacity to facilitate the integration of tNGS into national tuberculosis control programs [45,46].

Future studies combining prospective clinical data, functional analyses of uncharacterized variants, and large-scale surveillance will be essential to fully harness the potential of targeted sequencing for tuberculosis control and patient care.

## Figures and Tables

**Table 1:** Summary of resistance-associated variants (RVs) and uncharacterized variants (UVs) detected by the Deeplex^®^-MycTB assay among 68 sequenced clinical isolates.

			Deeplex^®^-MycTB Assay	
Drug Type	Drug	Locus	Number of Samples with RVs	Number of Samples with UVs
FIRST-LINE	RIF	*rpoB*	40	0
	INH	*katG*	42	0
		*fabG1*	16	0
		*ahpC*	0	0
		*inhA*	0	0
	PZA	*pncA*	18	25
	EMB	*embB*	29	8
GROUP A/B (XDR)	FQ	*gyrA*	14	0
		*gyrB*	5	0
	LIN	*rplC*	0	0
		*rrl*	0	3
	BDQ/CFZ	*Rv0678*	0	0
GROUP C	AMI	*rrs*	11	0
	SM	*gidB*	0	16
		*rpsL*	21	0
		*Rrs*	11	1
	ETH	*ethA*	32	9
		*inhA*	0	0
		*fabG1*	16	0
OTHERS	KAN	*Eis*	1	0
	KAN/CAP	*rrs*	11	1
	CAP	*tlyA*	0	0

AB: antibiotics; RVs: drug resistance-associated variants; UVs: uncharacterized variants; RIF: rifampicin; INH: isoniazid; PZA: pyrazinamide; EMB: ethambutol; SM: streptomycin; FQ: fluoroquinolones; ETH: ethionamide; PTH: prothionamide; KAN: kanamycin; AMI: amikacin; CAP: capreomycin; LIN: linezolid; BDQ: bedaquiline; CFZ: clofazimine.

**Table 2:** Detailed prediction of drug resistance-associated variants by the Deeplex-MycTB assay for 68 sequenced clinical samples.

Gene	Genomic Position	Codon Change	AA Change	Number of Samples	Drug
*embB*	4247429	ATG306GTG	M306V	10	EMB
	4248002	CAG497AAG	Q497K	9	
	4247431	ATG306GATT	M306I	2	
	4247431	ATG306ATA	M306I	4	
	4247730	GGC406GCC	G406A	3	
	4247402	TCG297GCG	S297A	1	
*gyrA*	7582	GAC94GGC	D94G	4	FQ
	7570	GCG90GTG	A90V	3	
	7572	TCG91CCG	S91P	1	
	7581	GAC94AAC	D94N	1	
	7581	GAC94CAC	D94H	1	
	7564	GGC88GCC	G88A	3	
	7581	GAC94TAC	D94Y	2	
*katG*	2155168	AGC315ACC	S315T	42	INH
*rpoB*	761155	TCG450TTG	S450L	31	RIF
	761155	TCG450TGG	S450W	2	
	761140	CAC445CTC	H445L	2	
	761139	CAC445TAC	H445Y	5	
	760314	GTC170TTC	V170F	2	
	761140	CAC445CGC	H445R	3	
*ethA*	4326930	inserC	FrSh	1	ETH
	4326210	delG	FrSh	6	
	4326640-6	delCACGTCG	FrSh	15	
	4327367	delT	FrSh	10	
*pncA*	2288852	inserC	FsSh	1	PZA
	2289218	GAC8GAG	D8E	5	
	2289010	delC	FrSh	6	
	2289252	A-11G	n/a	2	
	2288869	GTC125TTC	V125F	2	
	2288853-65	delACATCGACCTCAT	FrSh	1	
	2288794	inserC	FrSh	1	
	2289057	CCG62CTG	P62L	1	
*Rrs*	1472362	C517T	n/a	3	AMI, KAN, SM
	1472359	A514C	n/a	3	
	1473246	A1401G	n/a	4	
*rpsL*	781822	AAG88AAG	K88R	13	SM
	781687	AAG43AGG	K43R	8	SM
*fabG1*	1673425	C-15T	n/a	16	INH, EMB
*gyrB*	6620	GAC461AAC	D461N	2	FQ
	6750	GCG504GTG	A504V	1	FQ
	6738	ACC500AAC	T500N	2	FQ
*Eis*	2715346	C-14T	n/a	1	KAN

RIF: rifampicin; INH: isoniazid; PZA: pyrazinamide; EMB: ethambutol; SM: streptomycin; FQ: fluoroquinolones; ETH: ethionamide; KAN: kanamycin; AMI: amikacin; n/a: no statistically significant threshold reached, additional data required.

**Table 3:** Detailed prediction of uncharacterized variants by the Deeplex-MycTB assay for 68 sequenced clinical samples.

Gene	Genomic Position	Codon Change	AA Change	Number of Samples	Drug
*gidB*	4408072	CTA44CCA	L44P	1	SM
	4408105	delC	FrSh	2	
	4408137	TAC22TAA	Y22Stop	7	
	4407856	inserC	FrSh	6	
*ethA*	4326663	CAG271TAG	Q271Stop	5	ETH
	4327314	CGC54AGC	R54S	2	
	4326284	CTG397CCG	L397P	2	
*pncA*	2288754	CTG163GG	V163G	3	PZA
	2288817	ACG142AGG	T142R	14	
	2289097	GAC49TAC	D49Y	2	
	2289071	CAC57CAA	H57Q	2	
	2289027	TGC72TAC	C72Y	3	
	2288731	GCG171ACG	A171T	1	
*embB*	4247399	AAT296CAT	N296H	8	EMB
*rrl*	1476007	T2350G	n/a	2	AMI, KAN, SM
	1476251	T2594C	n/a	1	
*rrs*	1473123	A1278T	n/a	1	AMI, KAN, SM
	1472850	delT	deletion	1	
	1472857	delA	deletion	1	
	1472845	inserC	Insertion	1	
	1472860	inserG	Insertion	1	
	1473120	inserT	Insertion	1	
	1473396	inserG	Insertion	1	

PZA: pyrazinamide; EMB: ethambutol; SM: streptomycin; ETH: ethionamide; KAN: kanamycin; AMI: amikacin; n/a: no statistically significant threshold reached, additional data required; AA: amino acid.

**Table 4:** Concordance between phenotypic drug susceptibility testing (PDST) and targeted next-generation sequencing (tNGS) for rifampicin and isoniazid resistance in 68 Mycobacterium tuberculosis clinical isolates.

Resistance	Gene(s) with Mutations	Number of Samples with Mutations (tNGS)	Phenotypic DST Result	Concordance
Rifampicin (RIF)	rpoB	40	All 40 resistant	100%
Isoniazid (INH)	katG and/or fabG1	42	All 42 resistant	100%

**Table 5:** Detailed phylogeny for 40 MDR/pre-XDR clinical samples.

Sample ID	INH	RIF	FQ	LIN BDQ/CFZ	MDR	Pre-XDR	Spoligotype	SNP Phylogenetic Lineage
							Clade	Lineage
1							LAM1	L4.3
2							n/a	L4.3
5							T1	Other than H37Rv
6							n/a	Other than H37Rv
8							LAM9	L4.3
11							LAM9	L4.3
12							LAM9	L4.3
16							S	Other than H37Rv
17							Beijing	L2
18							Manu2	Other than H37Rv
19							LAM9	L4.3
20							T1	Other than H37Rv
21							Unknown	L2
22							LAM9	L4.3
24							T1	L4.3
26							T1	L4.3
27							T1	Other than H37Rv
29							LAM9	L4.3
33							T1	Other than H37Rv
34							T1	L4.3
35							T1	L4.3
37							LAM9	L4.3
40							LAM9	L4.3
41							LAM9	L4.3
43							H1	Other than H37Rv
45							Beijing	L2
46							S	Other than H37Rv
47							T1	Other than H37Rv
48							S	Other than H37Rv
52							Manu2	L2
53							Beijing	L2
54							S	Other than H37Rv
55							T1	L4.3
56							T1	Other than H37Rv
57							Beijing	L2
58							S	Other than H37Rv
60							n/a	Other than H37Rv
62							LAM9	L4.3
63							Beijing	L2
64							LAM9	L4.3
65							LAM9	L4.3
66							LAM9	L4.3
	42	40			40	5		

MDR: multidrug-resistant TB defined as resistance to at least rifampicin and isoniazid; Pre-XDR: pre-extensively drug-resistant TB defined as MDR-TB with additional resistance to any fluoroquinolone; RIF: rifampicin; INH: isoniazid; FQ: fluoroquinolones; LIN: linezolid; BDQ: bedaquiline; CFZ: clofazimine; n/a: no statistically significant threshold reached, additional data required. Color coding indicates resistance patterns: red = INH resistance, blue = RIF resistance, purple = fluoroquinolone resistance, yellow = MDR or pre-XDR status.

**Table 6:** Spoligotype clade/lineage correlation of *Mycobacterium tuberculosis* isolates from 68 sequenced clinical samples.

		Spoligotype Clade														
		LAM1	LAM4	LAM5	LAM9	T1	Beijing	Manu2	H1	H3	S	X	TH37-Rv	n/a	Unknown	NTM
Lineage	L4.3	1	1	1	18	8	*	*	*	*	*	*	*	2	1	*
	Other than H37rv	*	*	*	*	11	*	1	2	2	7	1	*	4	2	*
	L2	*	*	*	*	*	5	1	*	*	*	*	*	*	*	*
	Not detected	*	*	*	*	*	*	*	*	*	*	*	*	1	*	1
	No specific	*	*	*	*	*	*	*	*	*	*	*	1	*	*	*
	Total	1	1	1	18	19	5	1	2	2	7	1	1	7	3	1

n/a: no statistically significant threshold reached, additional data required; NTM: non-tuberculous Mycobacteria detected. ‘*’: indicates that no isolate was detected for the corresponding clade–lineage combination.

**Table 7:** Spoligotype clade/lineage correlation of *Mycobacterium tuberculosis* complex among 40 mdr-tb clinical samples from 68 sequenced clinical samples.

		Spoligotype Clade								
		LAM1	LAM9	T1	Beijing	Manu2	S	H1	n/a	Unknown
Lineage	%	2.5	30	28	10	5	12.5	2.5	5	2.5
	L4.3	1	12	5	*	*	*	*	1	1
	Other than H37rv	*	*	6	*	1	5	1	1	*
	L2	*	*	*	4	1	*	*	*	*
	Total	1	12	11	4	2	5	1	2	1

n/a: no statistically significant threshold reached, additional data required. ‘*’: indicates that no isolate was detected for the corresponding clade–lineage combination.

**Table 8:** The proportion of mixed infections on 68 sequenced clinical samples.

Mixed Infection	Number of Samples	%
Yes	11	16.2
No	57	83.8

## Data Availability

The original contributions presented in this study are included in the article/Supplementary Material. Further inquiries can be directed to the corresponding authors.

## Supplementary Materials

The following supporting information can be downloaded at: https://www.mdpi.com/article/10.3390/microorganisms13092163/s1. Table S1: Sequencing metrics by samples. Table S2: Median depth of coverage per gene targeted by the Deeplex-MycTB assay across samples. Table S3. Graphical summary of resistance prediction by the Deeplex -MycTB assay for 71 sequenced clinical samples. Table S4. Detailed phylogenetic analysis of 71 clinical samples by the Deeplex®-MycTB assay.

## Abbreviations

The following abbreviations are used in this manuscript:

TB	Tuberculosis
WHO	World Health Organization
NGS	Next-Generation Sequencing
HRM	High-Resolution Melting Curve
LJ	Löwenstein–Jensen
RRDR	Rifampicin Resistance-Determining Region
MTBC	Mycobacterium Tuberculosis Complex
XDR-TB	Extensively Drug-Resistant TB
Pre- XD	Pre-extensively Drug-Resistant TB
MDR-TB	Multidrug-Resistant
RIF	Rifampicin
AA	Amino Acid
INH	Isoniazid
PZA	Pyrazinamide
EMB	Ethambutol
SM	Streptomycin
FQ	Fluoroquinolones
ETH	Ethionamide
PTH	Prothionamide
KAN	Kanamycin
AMI	Amikacin
CAP	Capreomycin
LIN	Linezolid
BDQ	Bedaquiline
CF	Clofazimine
NTM	Non-tuberculous Mycobacteria
