# Supplementary material for: Characterization of Drug Resistance Mutations in Mycobacterium tuberculosis Isolates from Moroccan Patients Using Deeplex Targeted Next-Generation Sequencing
Source: Microorganisms. 2025 Sep 17;13(9):2163. doi: 10.3390/microorganisms13092163 (PMC12472841; doi:10.3390/microorganisms13092163)
Supplement: Supplementary file 1 [file microorganisms-13-02163-s001.zip › microorganisms-3753464-supplementary.pdf]

**Supplementary Table S1. Sequencing metrics by samples.**

| Sample ID | Sequencing Quality | Composite reference coverage | Median depth coverage | Min coverage | Max coverage |
|---|---|---|---|---|---|
| **1** | 2 | 100% | 1405x | 192x gyrA | 3961x eis |
| **2** | 2 | 100% | 798x | 92x rrs2 | 6248x eis |
| **3** | 2 | 100% | 2533x | 156x rrs2 | 3458x eis |
| **4** | 2 | 100% | 1981x | 212x rrs2 | 4041x inhA |
| **5** | 2 | 100% | 3483x | 173x rrs2 | 3469x inhA |
| **6** | 2 | 100% | 1784x | 135x rrs2 | 6060x eis |
| **7** | 2 | 100% | 3737x | 78x rrs2 | 21058x eis |
| **8** | 2 | 100% | 1106x | 92x rrs2 | 2660x eis |
| **9** | 2 | 100% | 2263x | 148x rrs2 | 11042x eis |
| **10** | 3 | 100% | 2287x | 80x rrs2 | 6687x eis |
| **11** | 2 | 100% | 2823x | 71x rrs2 | 24093x eis |
| **12** | 2 | 100% | 2874x | 298x rrs2 | 13530x eis |
| **13** | 2 | 100% | 3240x | 359x rrs2 | 15185x eis |
| **14** | 2 | 100% | 2011x | 144x rrs2 | 7653x eis |
| **15** | 3 | 100% | 30235x | 1076x rrs2 | 37582x eis |
| **16** | 1 | 100% | 1899x | 156x rrs2 | 8372x eis |
| **17** | 2 | 100% | 2306x | 66x rrs2 | 4030x eis |
| **18** | 2 | 100% | 5071x | 269x rrs2 | 16628x eis |
| **19** | 3 | 100% | 32724x | 503x rrs2 | 71049x eis |
| **20** | 2 | 100% | 3769x | 53x rrs2 | 5445x eis |
| **21** | 3 | 100% | 3835x | 260x rrs2 | 5181x embB |
| **22** | 2 | 100% | 6165x | 133x rrs2 | 9653x eis |
| **23** | 2 | 100% | 3406x | 153x rrs2 | 13289x eis |
| **24** | 3 | 100% | 11024x | 314x rrs2 | 9975x inhA |
| **25** | 3 | 100% | 5214x | 201x rrs2 | 10580x eis |

| | | | | | |
|---|---|---|---|---|---|
| **26** | 2 | 100% | 5263x | 213x rrs2 | 7505x eis |
| **27** | 3 | 100% | 13181x | 496x rrs2 | 22753x eis |
| **28** | 3 | 100% | 9769x | 638x rrs2 | 13089x rpoB2 |
| **29** | 3 | 100% | 5086x | 341x rrs2 | 11578x eis |
| **30** | 3 | 100% | 6628x | 531x rrs2 | 19388x eis |
| **31** | 3 | 100% | 7924x | 322x rrs2 | 14913x eis |
| **32** | 3 | 100% | 4516x | 302x rrs2 | 7514x eis |
| **33** | 3 | 100% | 4449x | 262x rrs2 | 15215x eis |
| **34** | 3 | 100% | 11209x | 270x rrs2 | 9022x rpoB1 |
| **35** | 3 | 100% | 8504x | 431x rrs2 | 34742x eis |
| **36** | 2 | 100% | 2786x | 160x rrs2 | 13109x eis |
| **37** | 2 | 100% | 5357x | 268x rss2 | 10495x eis |
| **38** | 3 | 100% | 7081x | 301x gyrA | 11564x rrl |
| **39** | 3 | 100% | 8105x | 248x rrs2 | 9582x eis |
| **40** | 2 | 100% | 2170x | 73x gyrA | 1837x embB |
| **41** | 2 | 100% | 3663x | 27x rrs2 | 4828x eis |
| **42** | 1 | 100% | 1833x | 42x rrs2 | 1856x eis |
| **43** | 2 | 100% | 2052x | 94x rrs2 | 1670x eis |
| **44** | NTM | 62,20% | 12x | 0x | 5499x |
| **45** | 3 | 100% | 6147x | 204x rrs2 | 4751x rpoB2 |
| **46** | 2 | 100% | 1322x | 55x gyrA | 1852x inhA |
| **47** | 2 | 100% | 854x | 55x rrs2 | 5563x eis |
| **48** | 2 | 100% | 1187x | 115x rrs2 | 2922x rrs1 |
| **49** | ND | 100% | 38x | 0x | 637x rrs1 |
| **50** | 1 | 99.9% | 875x | 79x rrs2 | 3869x rrs1 |
| **51** | ND | 89,50% | 0x | 0x | 20x eis |
| **52** | 2 | 100% | 849x | 56x rrs2 | 1970x rrs1 |
| **53** | 2 | 99,90% | 604x | 55x rrs2 | 898x rrl |
| **54** | 2 | 100% | 1832x | 126x rrs2 | 4080x eis |
| **55** | 2 | 100% | 2272x | 221x rrs2 | 11997x eis |

| | | | | | |
|---|---|---|---|---|---|
| **56** | 1 | 99.90% | 383x | 23x rrs2 | 1207x eis |
| **57** | 2 | 100% | 1382x | 113x rrs2 | 2338x rrs1 |
| **58** | 2 | 99.90% | 831x | 51x gyrA | 1617x rrs1 |
| **59** | 1 | 99.90% | 617x | 29x rrs2 | 2043x eis |
| **60** | 1 | 99,80% | 267x | 19x rrs2 | 446x rrl |
| **61** | 2 | 100% | 847x | 40x rrs2 | 1449x eis |
| **62** | 1 | 99.90% | 713x | 51x rrs2 | 7564x eis |
| **63** | 1 | 100% | 885x | 60x rrs2 | 1478x rrl |
| **64** | 2 | 100% | 1013x | 105x rrs2 | 2576x eis |
| **65** | 1 | 100% | 1859x | 32x rrs2 | 2057x eis |
| **66** | 2 | 100% | 1508x | 159x rrs2 | 3434x eis |
| **67** | 2 | 99.90% | 782x | 98x rrs2 | 1615x rrl |
| **68** | 2 | 100% | 1122x | 115x rrs2 | 4352x eis |
| **69** | 2 | 100% | 1669x | 100x rrs2 | 4974x eis |
| **70** | 3 | 93,30% | 2298x | 0x rrs1 | 10692x rrl |
| **71** | 1 | 99,90% | 760x | 16x rrs2 | 1148 gyrA |
| **Average** | | 99.21% | **3950,4x** | | |
| **Median** | | | **2263x** | | |

**1:** All resistance-associated positions in database with enough data to identify mutations from 80-100%; **2:** All resistance-associated positions in database with enough data to identify mutations from 10-100%; **3:** All resistance-associated positions in database with enough data to identify mutations from 3-100%; **ND:** Mycobacteria not detected; **NTM:** Nontuberculous mycobacteria detected; **Composite reference coverage**: coverage breadth over the concatenated reference sequences associated with drug resistance; **Median depth of coverage**: median of average read depths among reference sequences associated with drug resistance; **(min x, max x)**: minimal/maximal average coverage depth among the targets.

**Supplementary Table S2. Median depth of coverage per gene targeted by the Deeplex-MycTB assay across samples.**

| Sample ID | Median depth coverage | *rpob1* | *rpoB2* | *katG* | *fabG1* | *ahpC* | *inhA* | *pncA* | *embB* | *gidB* | *rpsL* | *gyrA* | *gyrB* | *ethA* | *eis* | *rrs2* | *rrs1* | *tlyA* | *rplC* | *rrl* | *Rv0678* |
|---|---|---|---|---|---|---|---|---|---|---|---|---|---|---|---|---|---|---|---|---|---|
| 1 | 1405x | 2776 | 2007 | 1044 | 1523 | 1106 | 1333 | 1093 | 696 | 533 | 1567 | 192 | 1140 | 522 | 3961 | 271 | 1082 | 288 | 1120 | 1241 | 673 |
| 2 | 798x | 4699 | 2672 | 621 | 2081 | 1790 | 3167 | 1089 | 852 | 570 | 1908 | 427 | 691 | 257 | 6248 | 92 | 202 | 179 | 1164 | 606 | 556 |
| 3 | 2533x | 3413 | 3282 | 1336 | 2009 | 1192 | 2977 | 1651 | 1352 | 873 | 1434 | 667 | 2071 | 718 | 3457 | 156 | 738 | 387 | 820 | 1230 | 975 |
| 4 | 1981x | 4040 | 3991 | 1745 | 2842 | 2240 | 3821 | 2482 | 1849 | 1201 | 2946 | 644 | 1533 | 690 | 3936 | 211 | 769 | 542 | 1418 | 1471 | 1574 |
| 5 | 3483x | 3197 | 3289 | 1313 | 1519 | 1584 | 3463 | 2548 | 2648 | 1156 | 1293 | 451 | 2686 | 724 | 2693 | 172 | 1064 | 355 | 646 | 1997 | 1172 |
| 6 | 1784x | 3365 | 3012 | 1149 | 2298 | 1398 | 2236 | 2029 | 1363 | 734 | 1969 | 744 | 1459 | 650 | 6065 | 134 | 596 | 306 | 1335 | 1431 | 1118 |
| 7 | 3737x | 10362 | 5959 | 1106 | 4917 | 2432 | 4132 | 2147 | 1489 | 523 | 3908 | 1630 | 2822 | 515 | 21057 | 78 | 376 | 50 | 3882 | 1911 | 1164 |
| 8 | 1106x | 1214 | 2659 | 1744 | 834 | 758 | 2096 | 1413 | 1504 | 746 | 538 | 113 | 987 | 820 | 922 | 92 | 1187 | 562 | 264 | 1249 | 900 |
| 9 | 2263x | 7595 | 4645 | 1521 | 3482 | 1748 | 3982 | 2916 | 1940 | 966 | 2784 | 1177 | 1755 | 669 | 11042 | 148 | 1150 | 443 | 2403 | 1805 | 1330 |
| 10 | 2287x | 6106 | 5027 | 2593 | 2695 | 1761 | 5190 | 3226 | 3629 | 1342 | 2225 | 594 | 1909 | 1111 | 6687 | 80 | 373 | 616 | 1432 | 911 | 1651 |
| 11 | 2823x | 16537 | 7683 | 1760 | 7298 | 2345 | 6024 | 3802 | 2909 | 928 | 5297 | 2853 | 2151 | 992 | 24093 | 71 | 50 | 50 | 5559 | 855 | 2227 |
| 12 | 2874x | 10598 | 6343 | 2792 | 4714 | 2641 | 5399 | 3819 | 3017 | 1529 | 4144 | 1843 | 2166 | 1082 | 13529 | 288 | 1555 | 642 | 3287 | 3714 | 1954 |
| 13 | 3240x | 13750 | 7331 | 2056 | 5001 | 2966 | 5492 | 3620 | 2619 | 1188 | 4925 | 2170 | 2572 | 950 | 15184 | 358 | 2051 | 447 | 3737 | 5927 | 1818 |
| 14 | 2011x | 5954 | 4257 | 1648 | 2555 | 1292 | 3098 | 2167 | 1720 | 822 | 1882 | 883 | 1558 | 776 | 7653 | 144 | 1680 | 367 | 1538 | 2788 | 1087 |
| 15 | 30235x | 32270 | 22477 | 7923 | 15722 | 7531 | 13911 | 11203 | 8465 | 4262 | 13361 | 5707 | 8301 | 3624 | 37582 | 1075 | 9740 | 1671 | 10495 | 18630 | 6705 |
| 16 | 1899x | 7408 | 4759 | 893 | 3071 | 1827 | 3071 | 1923 | 1361 | 450 | 2447 | 1112 | 1523 | 541 | 8372 | 156 | 862 | 180 | 2064 | 3180 | 1169 |
| 17 | 2306x | 3186 | 2866 | 918 | 1654 | 1334 | 1502 | 1384 | 1045 | 381 | 1180 | 695 | 1926 | 630 | 4030 | 66 | 297 | 150 | 980 | 780 | 806 |
| 18 | 5071x | 12810 | 6030 | 2120 | 5114 | 2998 | 4429 | 3329 | 2861 | 1086 | 4447 | 2388 | 3848 | 1059 | 16627 | 269 | 1794 | 470 | 4284 | 3777 | 2282 |
| 19 | 32724x | 52541 | 25184 | 6032 | 22824 | 8755 | 18514 | 11911 | 9493 | 2962 | 18369 | 9754 | 7676 | 3520 | 71049 | 503 | 2615 | 1291 | 16484 | 8377 | 7250 |
| 20 | 3769x | 4426 | 3018 | 805 | 2143 | 936 | 1835 | 1513 | 1380 | 408 | 1361 | 798 | 738 | 579 | 5444 | 53 | 256 | 243 | 1369 | 824 | 758 |
| 21 | 3835x | 3571 | 3104 | 3044 | 1630 | 1498 | 2672 | 3516 | 5181 | 2079 | 1405 | 637 | 2963 | 1491 | 3215 | 206 | 1321 | 1030 | 958 | 2260 | 1430 |
| 22 | 6165x | 6683 | 4871 | 2009 | 2993 | 1848 | 4103 | 3352 | 2093 | 1281 | 2326 | 1378 | 2428 | 942 | 9653 | 133 | 829 | 510 | 1920 | 1430 | 1432 |
| 23 | 3406x | 9018 | 6056 | 2368 | 4982 | 2505 | 4856 | 4430 | 3291 | 1729 | 4273 | 2153 | 2585 | 1041 | 13289 | 153 | 815 | 768 | 3774 | 1662 | 2231 |
| 24 | 11024x | 7161 | 8411 | 4802 | 4761 | 4931 | 9975 | 7075 | 6174 | 4489 | 4053 | 1266 | 5143 | 2814 | 8116 | 314 | 2413 | 1845 | 2307 | 3628 | 3123 |
| 25 | 5214x | 8784 | 7961 | 3466 | 4305 | 2609 | 7183 | 5932 | 4936 | 2246 | 3397 | 1524 | 3934 | 1506 | 10579 | 201 | 1767 | 998 | 2439 | 2624 | 2521 |

| | | | | | | | | | | | | | | | | | | | | | |
|---|---|---|---|---|---|---|---|---|---|---|---|---|---|---|---|---|---|---|---|---|---|
| 26 | 5263x | 6655 | 4165 | 1386 | 3215 | 2017 | 3297 | 2701 | 1909 | 1087 | 3623 | 1570 | 2052 | 735 | 7504 | 213 | 826 | 405 | 2412 | 1756 | 1716 |
| 27 | 13181x | 17172 | 9987 | 4337 | 9658 | 5545 | 7924 | 7295 | 5667 | 3324 | 10553 | 3797 | 4435 | 2197 | 22753 | 496 | 2145 | 1549 | 7299 | 3795 | 3908 |
| 28 | 9769x | 10544 | 13089 | 5824 | 3968 | 4467 | 11763 | 11635 | 6477 | 3938 | 4437 | 1341 | 7594 | 2100 | 7619 | 638 | 4565 | 1145 | 2091 | 9715 | 4214 |
| 29 | 5086x | 10308 | 8132 | 4278 | 5107 | 2385 | 5705 | 4087 | 4603 | 2477 | 4027 | 1686 | 3929 | 2228 | 11577 | 341 | 2947 | 1169 | 2932 | 3763 | 2691 |
| 30 | 6628x | 15770 | 10483 | 4311 | 7921 | 4150 | 7964 | 6778 | 5283 | 2949 | 6612 | 3331 | 5042 | 1854 | 19388 | 531 | 2758 | 1243 | 5297 | 5138 | 3630 |
| 31 | 7924x | 11715 | 6018 | 2277 | 6230 | 3805 | 5234 | 4068 | 3116 | 1932 | 6098 | 2124 | 2532 | 1202 | 14913 | 332 | 1120 | 731 | 4446 | 2763 | 2255 |
| 32 | 4516x | 7063 | 6731 | 3407 | 3591 | 2245 | 5090 | 4547 | 4117 | 2259 | 2836 | 1192 | 3547 | 1602 | 7514 | 302 | 2606 | 1071 | 1994 | 4228 | 2336 |
| 33 | 4449x | 12724 | 9256 | 3269 | 7091 | 3331 | 6382 | 6654 | 5861 | 2654 | 5205 | 2640 | 3368 | 1745 | 15214 | 262 | 1590 | 1078 | 4574 | 3246 | 3755 |
| 34 | 11209x | 9022 | 8729 | 3134 | 4186 | 2825 | 6303 | 6016 | 5219 | 2109 | 3375 | 1250 | 4096 | 1449 | 7943 | 270 | 3004 | 735 | 2142 | 6340 | 3185 |
| 35 | 8504x | 25190 | 14643 | 5116 | 10912 | 5320 | 11133 | 9636 | 7429 | 3552 | 9228 | 5154 | 6386 | 2497 | 34742 | 431 | 2857 | 1503 | 7896 | 5019 | 4721 |
| 36 | 2786x | 10528 | 6076 | 2371 | 5196 | 2389 | 5147 | 4085 | 2950 | 1219 | 4821 | 2441 | 2118 | 947 | 13108 | 160 | 664 | 515 | 3622 | 1346 | 2144 |
| 37 | 5357x | 7838 | 4040 | 1792 | 3198 | 1505 | 3495 | 2600 | 2326 | 1045 | 2226 | 1441 | 1678 | 660 | 10494 | 267 | 2657 | 545 | 2249 | 4316 | 1385 |
| 38 | 7081x | 4933 | 5549 | 4179 | 1434 | 1617 | 5182 | 4058 | 5260 | 2690 | 1033 | 300 | 3456 | 2107 | 3694 | 351 | 7454 | 1501 | 599 | 11564 | 1494 |
| 39 | 8105x | 7599 | 6178 | 3100 | 3945 | 1901 | 4522 | 3916 | 3145 | 1812 | 2503 | 1176 | 2676 | 1277 | 9582 | 249 | 3500 | 994 | 2265 | 3782 | 1837 |
| 40 | 2170x | 1088 | 1656 | 1608 | 496 | 554 | 1374 | 1263 | 1837 | 1114 | 323 | 73 | 954 | 1275 | 781 | 102 | 1697 | 1018 | 158 | 1545 | 570 |
| 41 | 3663x | 4286 | 2876 | 816 | 1362 | 726 | 2153 | 1252 | 1221 | 527 | 1226 | 568 | 1168 | 659 | 4828 | 77 | 768 | 247 | 952 | 900 | 635 |
| 42 | 1833x | 1007 | 1426 | 843 | 901 | 574 | 665 | 909 | 857 | 414 | 465 | 204 | 771 | 552 | 1855 | 42 | 571 | 240 | 416 | 707 | 514 |
| 43 | 2052x | 1337 | 1558 | 1325 | 860 | 851 | 1319 | 1174 | 1352 | 958 | 973 | 270 | 784 | 1102 | 1670 | 93 | 706 | 642 | 453 | 550 | 579 |
| 44 | 12x | | | | | | | | | | | | | | | | | | | | |
| 45 | 6147x | 2889 | 4751 | 2501 | 1464 | 1562 | 2975 | 3284 | 2884 | 1425 | 1234 | 408 | 3076 | 1496 | 2502 | 204 | 2331 | 871 | 696 | 3491 | 1489 |
| 46 | 1322x | 1030 | 1669 | 1523 | 848 | 1517 | 1851 | 1333 | 1666 | 1417 | 687 | 55 | 1127 | 1275 | 1163 | 132 | 1330 | 625 | 344 | 1772 | 915 |
| 47 | 854x | 2410 | 1510 | 769 | 1269 | 436 | 1434 | 1023 | 930 | 419 | 499 | 475 | 671 | 450 | 5563 | 55 | 1477 | 250 | 939 | 1289 | 472 |
| 48 | 1187x | 1168 | 1590 | 1017 | 758 | 525 | 1151 | 1038 | 848 | 605 | 528 | 187 | 1029 | 659 | 2368 | 115 | 2922 | 426 | 418 | 2185 | 447 |
| 49 | 38x | | | | | | | | | | | | | | | | | | | | |
| 50 | 875x | 503 | 595 | 394 | 238 | 144 | 882 | 361 | 405 | 157 | 112 | 228 | 748 | 308 | 1137 | 79 | 3869 | 110 | 177 | 2197 | 168 |
| 51 | 0x | | | | | | | | | | | | | | | | | | | | |
| 52 | 849x | 825 | 1076 | 1142 | 373 | 321 | 865 | 797 | 895 | 750 | 197 | 73 | 728 | 880 | 859 | 56 | 1970 | 597 | 164 | 1557 | 337 |
| 53 | 604x | 646 | 817 | 626 | 464 | 445 | 777 | 860 | 829 | 521 | 342 | 66 | 439 | 482 | 702 | 55 | 739 | 316 | 202 | 898 | 432 |
| 54 | 1832x | 2345 | 2397 | 1593 | 1640 | 991 | 1836 | 1532 | 1982 | 646 | 1524 | 541 | 1562 | 917 | 4079 | 125 | 1922 | 406 | 1168 | 2740 | 1418 |
| 55 | 2272x | 8676 | 4188 | 2332 | 3358 | 1538 | 3494 | 2979 | 2867 | 1330 | 2340 | 1430 | 1729 | 840 | 11996 | 211 | 1792 | 793 | 2762 | 3667 | 1499 |

| | | | | | | | | | | | | | | | | | | | | | |
|---|---|---|---|---|---|---|---|---|---|---|---|---|---|---|---|---|---|---|---|---|---|
| 56 | 383x | 569 | 559 | 325 | 372 | 219 | 355 | 409 | 344 | 205 | 264 | 158 | 312 | 177 | 1207 | 23 | 636 | 161 | 233 | 639 | 192 |
| 57 | 1382x | 651 | 1015 | 975 | 384 | 493 | 803 | 949 | 1723 | 809 | 346 | 131 | 1192 | 848 | 927 | 113 | 2337 | 635 | 226 | 1964 | 412 |
| 58 | 831x | 407 | 840 | 793 | 185 | 425 | 836 | 1073 | 991 | 721 | 217 | 51 | 677 | 455 | 520 | 68 | 1616 | 501 | 125 | 1601 | 348 |
| 59 | 617x | 1436 | 960 | 560 | 586 | 308 | 791 | 611 | 627 | 358 | 370 | 228 | 476 | 196 | 2043 | 29 | 466 | 228 | 397 | 478 | 301 |
| 60 | 267x | 266 | 297 | 216 | 137 | 111 | 211 | 246 | 276 | 168 | 133 | 48 | 221 | 150 | 299 | 19 | 362 | 106 | 84 | 445 | 146 |
| 61 | 847x | 1232 | 1303 | 1207 | 529 | 414 | 1030 | 1063 | 1407 | 800 | 309 | 112 | 733 | 755 | 1448 | 40 | 501 | 1002 | 294 | 846 | 535 |
| 62 | 713x | 3816 | 1880 | 330 | 1340 | 598 | 1396 | 703 | 400 | 193 | 846 | 427 | 585 | 197 | 7563 | 51 | 485 | 85 | 1059 | 1150 | 416 |
| 63 | 885x | 912 | 1183 | 1107 | 358 | 348 | 688 | 752 | 912 | 679 | 242 | 65 | 709 | 763 | 765 | 59 | 726 | 390 | 216 | 1477 | 480 |
| 64 | 1013x | 1487 | 1569 | 687 | 827 | 557 | 1177 | 998 | 848 | 367 | 597 | 324 | 856 | 445 | 2576 | 105 | 1886 | 303 | 651 | 2052 | 622 |
| 65 | 1859x | 1642 | 1535 | 392 | 1011 | 758 | 1516 | 910 | 688 | 245 | 820 | 228 | 530 | 251 | 2057 | 32 | 257 | 184 | 541 | 535 | 520 |
| 66 | 1508x | 2022 | 2292 | 1062 | 1120 | 801 | 1576 | 1436 | 1311 | 571 | 777 | 477 | 1301 | 693 | 3434 | 159 | 2976 | 455 | 848 | 3140 | 959 |
| 67 | 782x | 991 | 907 | 1154 | 427 | 344 | 736 | 780 | 898 | 902 | 328 | 130 | 683 | 870 | 1225 | 97 | 1580 | 791 | 262 | 1615 | 456 |
| 68 | 1122x | 1817 | 1849 | 599 | 1117 | 650 | 1325 | 1171 | 682 | 366 | 808 | 475 | 932 | 436 | 4352 | 114 | 607 | 267 | 863 | 1697 | 510 |
| 69 | 1669x | 2769 | 3358 | 1378 | 1770 | 1257 | 2591 | 1889 | 1928 | 644 | 914 | 443 | 1355 | 876 | 4973 | 100 | 1294 | 593 | 976 | 2157 | 937 |
| 70 | 2298x | 2239 | 4183 | 0 | 1269 | 1206 | 3010 | 3233 | 3680 | 1716 | 870 | 259 | 2113 | 1603 | 2733 | 400 | 7968 | 963 | 594 | 10691 | 1232 |
| 71 | 760x | 572 | 714 | 410 | 404 | 275 | 592 | 767 | 506 | 286 | 297 | 1148 | 606 | 227 | 1118 | 16 | 456 | 202 | 298 | 338 | 322 |

**Median depth of coverage**: median of average read depths among reference sequences associated with drug resistance; **(min x, max x)**: minimal/maximal average coverage depth among the target

**Supplementary Table S3. Graphical summary of resistance prediction by the Deeplex -MycTB assay for 71 sequenced clinical samples.**

| Sample ID | *rpoB* | *katG* | *fabG1* | *ahpC* | *inhA* | *pncA* | *embB* | *gidB* | *rpsL* | *rrs* | *gyrA* | *gyrB* | *inhA* | *fabG1* | *Eis* | *Rrs* | *tlyA* | *ethA* | *rplC* | *rrl* | *Rv0678* |
|---|---|---|---|---|---|---|---|---|---|---|---|---|---|---|---|---|---|---|---|---|---|
| 1 | | | | | | | | | | | | | | | | | | | | | |
| 2 | | | | | | | | | | | | | | | | | | | | | |
| 3 | | | | | | | | | | | | | | | | | | | | | |
| 4 | | | | | | | | | | | | | | | | | | | | | |
| 5 | | | | | | | | | | | | | | | | | | | | | |
| 6 | | | | | | | | | | | | | | | | | | | | | |
| 7 | | | | | | | | | | | | | | | | | | | | | |
| 8 | | | | | | | | | | | | | | | | | | | | | |
| 9 | | | | | | | | | | | | | | | | | | | | | |
| 10 | | | | | | | | | | | | | | | | | | | | | |
| 11 | | | | | | | | | | | | | | | | | | | | | |
| 12 | | | | | | | | | | | | | | | | | | | | | |
| 13 | | | | | | | | | | | | | | | | | | | | | |
| 14 | | | | | | | | | | | | | | | | | | | | | |
| 15 | | | | | | | | | | | | | | | | | | | | | |
| 16 | | | | | | | | | | | | | | | | | | | | | |
| 17 | | | | | | | | | | | | | | | | | | | | | |
| 18 | | | | | | | | | | | | | | | | | | | | | |
| 19 | | | | | | | | | | | | | | | | | | | | | |
| 20 | | | | | | | | | | | | | | | | | | | | | |
| 21 | | | | | | | | | | | | | | | | | | | | | |
| 22 | | | | | | | | | | | | | | | | | | | | | |
| 23 | | | | | | | | | | | | | | | | | | | | | |
| 24 | | | | | | | | | | | | | | | | | | | | | |
| 25 | | | | | | | | | | | | | | | | | | | | | |
| 26 | | | | | | | | | | | | | | | | | | | | | |

| | | | | | | | | | | | | | | | | | | | | | |
|---|---|---|---|---|---|---|---|---|---|---|---|---|---|---|---|---|---|---|---|---|---|
| 27 | | | | | | | | | | | | | | | | | | | | | |
| 28 | | | | | | | | | | | | | | | | | | | | | |
| 29 | | | | | | | | | | | | | | | | | | | | | |
| 30 | | | | | | | | | | | | | | | | | | | | | |
| 31 | | | | | | | | | | | | | | | | | | | | | |
| 32 | | | | | | | | | | | | | | | | | | | | | |
| 33 | | | | | | | | | | | | | | | | | | | | | |
| 34 | | | | | | | | | | | | | | | | | | | | | |
| 35 | | | | | | | | | | | | | | | | | | | | | |
| 36 | | | | | | | | | | | | | | | | | | | | | |
| 37 | | | | | | | | | | | | | | | | | | | | | |
| 38 | | | | | | | | | | | | | | | | | | | | | |
| 39 | | | | | | | | | | | | | | | | | | | | | |
| 40 | | | | | | | | | | | | | | | | | | | | | |
| 41 | | | | | | | | | | | | | | | | | | | | | |
| 42 | | | | | | | | | | | | | | | | | | | | | |
| 43 | | | | | | | | | | | | | | | | | | | | | |
| 45 | | | | | | | | | | | | | | | | | | | | | |
| 46 | | | | | | | | | | | | | | | | | | | | | |
| 47 | | | | | | | | | | | | | | | | | | | | | |
| 48 | | | | | | | | | | | | | | | | | | | | | |
| 50 | | | | | | | | | | | | | | | | | | | | | |
| 51 | | | | | | | | | | | | | | | | | | | | | |
| 52 | | | | | | | | | | | | | | | | | | | | | |
| 53 | | | | | | | | | | | | | | | | | | | | | |
| 54 | | | | | | | | | | | | | | | | | | | | | |
| 55 | | | | | | | | | | | | | | | | | | | | | |
| 56 | | | | | | | | | | | | | | | | | | | | | |
| 57 | | | | | | | | | | | | | | | | | | | | | |
| 58 | | | | | | | | | | | | | | | | | | | | | |
| 60 | | | | | | | | | | | | | | | | | | | | | |
| 61 | | | | | | | | | | | | | | | | | | | | | |

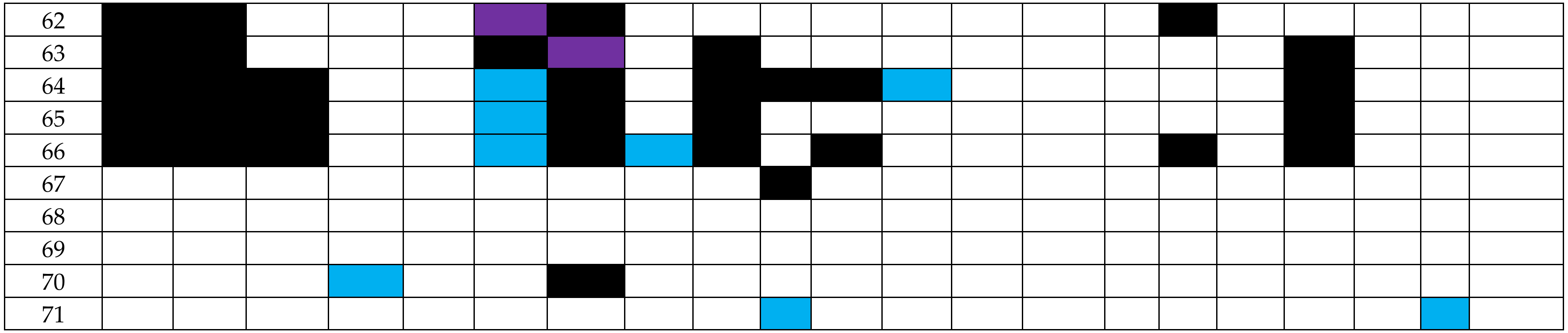


**Black frame**: Drug resistance associated variant/s; **Blue frame**: Uncharacterized variant/s; **Purple frame**: Uncharacterized variant/s in addition to drug resistance-associated variant/s.

**Supplementary Table S4. Detailed phylogenetic analysis of 71 clinical samples by the Deeplex®-MycTB assay**

| Sample ID | hsp-65 based species identification | | | | Spoligotype | | SNP phylogenetic lineage | Mixed infection |
|---|---|---|---|---|---|---|---|---|
| | Av.coverage depth | Consensus length | Identity | Best match | Av.coverage depth | Clade | Lineage | Detected |
| **1** | 2665x | 400 | 100% | *MTBC* | 1751x | LAM1 | L4.3 | No |
| **2** | 4285x | 400 | 100% | *MTBC* | 690x | n/a | L4.3 | No |
| **3** | 1556x | 400 | 100% | *MTBC* | 809x | UNKW | Other than Hrv37 | No |
| **4** | 3274x | 400 | 100% | *MTBC* | 613x | LAM5 | L4.3 | No |
| **5** | 1174x | 400 | 100% | *MTBC* | 613x | T1 | Other than Hrv37 | No |
| **6** | 2185x | 400 | 100% | *MTBC* | 323x | n/a | Other than Hrv37 | No |
| **7** | 6990x | 400 | 100% | *MTBC* | 1004x | H3 | Other than Hrv37 | No |
| **8** | 410x | 400 | 100% | *MTBC* | 307x | LAM9 | L4.3 | No |
| **9** | 3727x | 400 | 100% | *MTBC* | 323x | T1 | Other than Hrv37 | No |
| **10** | 2355x | 400 | 100% | *MTBC* | 884x | LAM9 | L4.3 | No |
| **11** | 8312x | 400 | 100% | *MTBC* | 495x | LAM9 | L4.3 | No |
| **12** | 4885x | 400 | 100% | *MTBC* | 1320x | LAM9 | L4.3 | No |
| **13** | 6518x | 400 | 100% | *MTBC* | 736x | T1 | L4.3 | Yes |
| **14** | 2281x | 400 | 100% | *MTBC* | 305x | T1 | Other than Hrv37 | No |
| **15** | 16042x | 400 | 100% | *MTBC* | 1419x | T1 | L4.3 | No |
| **16** | 3500x | 400 | 100% | *MTBC* | 258x | S | Other than Hrv37 | No |
| **17** | 1634x | 400 | 100% | *MTBC* | 499x | Beijing | L2 | No |
| **18** | 6165x | 400 | 100% | *MTBC* | 983x | Manu2 | Other than Hrv37 | Yes |
| **19** | 28096x | 400 | 100% | *MTBC* | 2249x | LAM9 | L4.3 | No |
| **20** | 2563x | 400 | 100% | *MTBC* | 194x | T1 | Other than Hrv37 | No |
| **21** | 806x | 400 | 100% | *MTBC* | 3286x | UNKW | L2 | No |
| **22** | 2721x | 400 | 100% | *MTBC* | 412x | LAM9 | L4.3 | No |
| **23** | 4943x | 400 | 100% | *MTBC* | 780x | H3 | Other than Hrv37 | No |
| **24** | 4425x | 400 | 100% | *MTBC* | 855x | T1 | L4.3 | No |

| | | | | | | | | |
|---|---|---|---|---|---|---|---|---|
| **25** | 3020x | 400 | 100% | *MTBC* | 718x | T1 | Other than Hrv37 | Yes |
| **26** | 4252x | 400 | 100% | *MTBC* | 308x | T1 | L4.3 | Yes |
| **27** | 11955x | 400 | 100% | *MTBC* | 1333x | T1 | Other than Hrv37 | Yes |
| **28** | 2851x | 400 | 100% | *MTBC* | 766x | LAM4 | L4.3 | No |
| **29** | 3983x | 400 | 100% | *MTBC* | 780x | LAM9 | L4.3 | No |
| **30** | 7244x | 400 | 100% | *MTBC* | 1609x | UNKW | Other than Hrv37 | No |
| **31** | 8544x | 400 | 100% | *MTBC* | 884x | S | Other than Hrv37 | No |
| **32** | 2859x | 400 | 100% | *MTBC* | 576x | T1 | L4.3 | Yes |
| **33** | 5801x | 400 | 100% | *MTBC* | 429x | T1 | Other than Hrv37 | Yes |
| **34** | 2855x | 400 | 100% | *MTBC* | 301x | T1 | L4.3 | No |
| **35** | 10840x | 400 | 100% | *MTBC* | 805x | T1 | L4.3 | Yes |
| **36** | 4789x | 400 | 100% | *MTBC* | 485x | T1 | Other than Hrv37 | No |
| **37** | 2993x | 400 | 100% | *MTBC* | 529x | LAM9 | L4.3 | No |
| **38** | 886x | 400 | 100% | *MTBC* | 1012x | LAM9 | L4.3 | No |
| **39** | 2401x | 400 | 100% | *MTBC* | 750x | LAM9 | L4.3 | No |
| **40** | 244x | 400 | 100% | *MTBC* | 820x | LAM9 | L4.3 | No |
| **41** | 2116x | 400 | 100% | *MTBC* | 362x | LAM9 | L4.3 | No |
| **42** | 535x | 400 | 100% | *MTBC* | 160x | S | Other than Hrv37 | No |
| **43** | 1270x | 400 | 100% | *MTBC* | 590x | H1 | Other than Hrv37 | No |
| **44** | 1653x | 400 | | M IntraCellular | - | - | - | - |
| **45** | 823x | 400 | 100% | *MTBC* | 1322x | Beijing | L2 | No |
| **46** | 696x | 400 | 100% | *MTBC* | 353x | S | Other than Hrv37 | No |
| **47** | 705x | 399 | 100% | *MTBC* | 517x | T1 | Other than Hrv37 | No |
| **48** | 493x | 399 | 100% | *MTBC* | 221x | S | Other than Hrv37 | No |
| **49** | 14x | 396 | 100% | *MTBC* | 89x | LAM9 | Not detected | ** |
| **50** | 86x | 397 | 100% | *MTBC* | 404x | T1 | Other than Hrv37 | No |
| **51** | 5x | 394 | 99,30% | *MTBC* | 14x | n/a | ND | ** |
| **52** | 167x | 399 | 100% | *MTBC* | 379x | Manu2 | L2 | Yes |
| **53** | 337x | 399 | 100% | *MTBC* | 1272x | Beijing | L2 | No |
| **54** | 1873x | 400 | 100% | *MTBC* | 494x | S | Other than Hrv37 | No |

| | | | | | | | | |
|---|---|---|---|---|---|---|---|---|
| **55** | 2953x | 400 | 100% | *MTBC* | 6596x | T1 | L4.3 | No |
| **56** | 209x | 400 | 100% | *MTBC* | 1086x | T1 | Other than Hrv37 | No |
| **57** | 204x | 395 | 100% | *MTBC* | 1250x | Beijing | L2 | No |
| **58** | 133x | 399 | 100% | *MTBC* | 133x | S | Other than Hrv37 | No |
| **59** | 460x | 400 | 100% | *MTBC* | 1265x | n/a | L4.3 | No |
| **60** | 103x | 397 | 100% | *MTBC* | 65x | n/a | Other than Hrv37 | Yes |
| **61** | 347x | 400 | 100% | *MTBC* | 832x | X1 | Other than Hrv37 | No |
| **62** | 2122x | 400 | 100% | *MTBC* | 546x | LAM9 | L4.3 | Yes |
| **63** | 179x | 397 | 100% | *MTBC* | 2828x | Beijing | L2 | No |
| **64** | 514x | 400 | 100% | *MTBC* | 280x | LAM9 | L4.3 | No |
| **65** | 1288x | 400 | 100% | *MTBC* | 251x | LAM9 | L4.3 | No |
| **66** | 706x | 400 | 100% | *MTBC* | 322x | LAM9 | L4.3 | No |
| **67** | 277x | 400 | 100% | *MTBC* | 2105x | n/a | Other than Hrv37 | No |
| **68** | 785x | 400 | 100% | *MTBC* | 528x | LAM9 | L4.3 | No |
| **69** | 1248x | 400 | 100% | *MTBC* | 291x | n/a | Other than Hrv37 | No |
| **70** | 645x | 400 | 100% | *MTBC* | 1766x | H1 | Other than Hrv37 | No |
| **71** | 233x | 400 | 100% | *MTBC* | 163x | T-H37Rv | No specific lineage | No |

**n/a**: No statistically significant threshold reached/additional data required; **ND:** Mycobacteria not detected.
